# Breaking barriers: ten essential steps to achieve gender equality in academia through scientific societies

**DOI:** 10.1038/s44185-025-00105-6

**Published:** 2025-10-01

**Authors:** Ana Genua-Olmedo, Sílvia Poblador, Judit Lecina-Diaz, María Mar Sánchez-Montoya, Pablo Rodríguez-Lozano, Mireia Bartrons, Elena Hernández-del Amo, María Anton-Pardo, Núria Catalán, Ada Pastor, Miguel Cañedo-Argüelles, Anna Freixa, Susana Bernal

**Affiliations:** 1 Grupo Gender & Science, Asociación Ibérica de Limnología, Mislata, Valencia Spain; 2 Tragsatec, Madrid, Spain; 3 CREAF, Cerdanyola del Vallès, Barcelona, Spain; 4 Technical University of Munich, TUM School of Life Sciences, Ecosystem Dynamics and Forest Management Group, Freising, Germany; 5 Department of Biodiversity, Ecology and Evolution, Complutense University of Madrid, Madrid, Spain; 6 Departamento de Ecología, Universidad Autónoma de Madrid, Madrid, Spain; 7 Aquatic Ecology Group, University of Vic - Central University of Catalonia (UVic-UCC), Vic, Spain; 8 Departament de Genètica i Microbiologia, Universitat Autònoma de Barcelona, Barcelona, Spain; 9 Institut Cavanilles de Biodiversitat i Biologia Evolutiva, Universitat de València, València, Spain; 10 Centre d’Estudis Avançats de Blanes, CEAB-CSIC, Blanes, Spain; 11 Group of Continental Aquatic Ecology Research (GRECO), Institute of Aquatic Ecology, University of Girona, Girona, Spain; 12 FEHM-Lab (Freshwater Ecology, Hydrology and Management), SHE-2, Institute of Environmental Assessment and Water Research (IDAEA), CSIC, Barcelona, Spain; 13 Catalan Institute for Water Research (ICRA-CERCA), Girona, Spain

**Keywords:** Biodiversity, Biogeography

## Abstract

The gender gap in STEM is a persistent global issue. Scientific societies can address this by promoting gender equity through collaboration, advocacy, and leadership. This study analyses gender representation on executive boards and the presence of gender groups in freshwater societies. Drawing on a decade of experience, it proposes ten actionable steps, highlights obstacles, and calls on societies to actively foster gender equity within academia and beyond.

## Introduction

The gender gap in academia is widespread and persistent worldwide, being particularly significant in Science, Technology, Engineering, and Mathematics (STEM) disciplines^1^. At present, women represent only 32% of researchers worldwide, reflecting just a modest increase of 4% from 2010 to 2018 (UNESCO, 2024). A significant drop in women's representation occurs as academic careers advance. For example, while Europe is close to gender parity at the doctoral level (48% of doctoral graduates are women), only 33% of women hold permanent research positions, and their representation falls below 26% at the highest academic level such as full professors or board directors^2^. These numbers highlight that, despite achieving similar levels of education, women still face more barriers than men when pursuing the same career path. This pattern has been described by the *Leaky Pipeline* metaphor, a phenomenon that describes the progressive loss of women at different points along the career trajectory^3^, and applies not only to women but also to other marginalized groups. The pervasive slowdown of women's careers creates a vicious circle: their underrepresentation in decision-making roles at the highest levels of politics and management in academia hinders efforts to address gender inequality effectively. This negative feedback loop deepens the structural segregation of women in academia, making true gender parity increasingly difficult to achieve.

While gender gaps have been mostly quantified between men and women, growing evidence shows that these inequalities also exist for other genders and marginalized groups. These include people marginalized by age, race, ethnicity, gender identity, sexual orientation, religion, and physical or mental disabilities^3,4^. The concept of the *Leaky Pipeline* applies to all these minoritised groups, many of whom experience multiple and intersecting forms of discrimination that can intensify their exclusion^5^. This perspective, known as intersectionality (https://eige.europa.eu/publications-resources/thesaurus), recognizes that people do not experience inequality based on a single factor (such as gender), but through the interactions of multiple aspects of their identity. For instance, gender gaps can vary significantly among women depending on factors such as race or income. Unfortunately, data on the representation of other genders (e.g. non-binary, cisgender, transgender, genderqueer, agender, and genderfluid) and marginalized groups in scientific societies remains very limited. Although our analysis focuses specifically on gender, and more precisely on women, many of the insights and recommendations provided in this paper are relevant and beneficial for advancing gender diversity and inclusion more broadly.

The *Leaky Pipeline* metaphor implies that the decline of women (and other minoritised groups) in academia is a passive process within an otherwise functioning system, and thus, overlooks the exclusionary mechanisms that actively push women out of their academic careers^4^. However, gender studies highlight that the reasons driving women away from academia are related to less research proposal submissions, colleague recommendations, visibility^6^, and recognition (i.e. promotions and awards) as well as unfavorable hiring conditions and explicit and implicit patriarchal violence. Based on this evidence, some authors have proposed that rather than a *Leaky Pipeline*, the situation experienced by women and other scientists from marginalized groups is better illustrated by the metaphor of the *Hostile Obstacle Course*^4^. In this analogy, their withdrawal from academia does not result from a personal decision in a neutral environment, but the result of many visible and invisible barriers encountered throughout their careers. These many obstacles range from very subtle (e.g. microaggressions, patriarchal behaviors) to very explicit (e.g. assault and sexual harassment), and can be visualized through the *Gender Equity Iceberg*^7^. The iceberg’s hidden part represents the most invisible and subtle obstacles, rooted in the core beliefs of our patriarchal society, that often go unnoticed but have a profound impact on the performance and wellbeing of women and other minoritised groups. Regardless of gender identity, we all perpetuate these unconscious biases, frequently underestimating their significance and damaging effects. In academia, these invisible obstacles can consist of being mistaken for administrative staff, not being recognized as the expert in a specific field, not getting the appropriate credit for their work, being excluded or ignored from meetings, not being invited to collaborate, being excluded from emails, experiencing hostility and obscene gestures, and receiving body remarks from male colleagues. Thus, there is an urgent need to make these obstacles visible, establish mechanisms to dismantle them, and actively engage the entire STEM community in rethinking how we build our academic environments. Applying the principles of justice, equity, diversity, and inclusion (JEDI) provides a necessary framework to drive this transformation (Box 1). By embedding these principles into our academic structures, we can foster a healthier, more respectful, kind, and inclusive environment - free from gender bias and all forms of minority-based discrimination - where every individual can thrive and contribute meaningfully to science.

Scientific societies emerged as early as in the XVII century, and were originally established to disseminate knowledge among scientists from different academic institutions, as well as to serve society by discussing and challenging new findings within specific disciplines^8^. Today, they play a pivotal role as platforms for transferring knowledge between academic institutions and citizens, while fostering community values and promoting supportive environments within academia^9^. Consequently, they can act as agents of change, boosting diversity and equity within the scientific community and society at large, and promoting transformative actions and programs that counterbalance mainstream patriarchal and non-inclusive behaviors. In the last decade, growing awareness of the underrepresentation of women and other minoritised groups in the professional environment, especially in science, has sparked internal discussion in many professional societies. Some of them have conducted demographic surveys to identify gender and other biases among members, as well as during conferences, which are benchmark events within scientific societies^9–13^. In some cases, this initial diagnosis has led to concrete recommendations and the implementation of targeted actions to improve gender equity^14,15^ (gender equity is the tool to achieve gender equality, see Box 1). Such actions include developing codes of conduct and philosophical statements grounded in JEDI principles, reviewing past exclusionary practices, empowering minoritised groups, and allocating resources to engage young researchers and reduce inequities (e.g. specific travel grants, mixer celebrations during conferences, establishment of new award categories)^9,10^. Therefore, scientific societies have significant potential to catalyze transformative changes, beyond individual actions, toward a more inclusive and equitable academia that can percolate beyond the limits of the scientific society itself.

This paper aims to encourage scientific societies to actively raise awareness and implement actions that advance gender equity in academia. Drawing from our ten years of experience as members of the Gender & Science group of the Iberian Society of Limnology (hereafter, G&S-AIL group), we believe that our learning processes, established workflows, and the challenges we have faced can be informative and provide insights to other scientific societies seeking to become agents of change towards a more inclusive and equitable academia. Although our experience has primarily focused on gender bias between men and women we believe that the learning processes and mechanisms proposed here can be equally relevant for fostering inclusivity and equality across the many other gender identities and other intersectional dimensions. These insights can help support not only women but also individuals from other minoritised groups. In this paper, we analyze gender biases in scientific societies and their boards, reporting the existence of gender committees within these societies (i.e. group of volunteer members devoted to promoting gender balance). We also consider the existence of JEDI committees within the boards that work for more diverse and egalitarian societies. We focus on executive boards and gender (or JEDI) committees because they represent, respectively, top-down and bottom-up organizational mechanisms for driving transformative change within scientific societies. We particularly focus on freshwater scientific societies as a case-study. Based on our experience, we then propose ten guiding steps, illustrated with specific actions and examples, aimed to help scientific societies and members implement initiatives that promote actions towards gender equality. Finally, we identify major obstacles to implementing these actions and outline future opportunities for strengthening gender balance and inclusiveness within scientific societies.

Box 1 Definitions - **Gender**: A social, psychological, and cultural construct distinct from biological sex, shaped through socialization and linked to self-identity. Gender includes identities such as masculine, feminine, transgender, non-binary, agender, and others^1^. This manuscript focuses on women due to data availability but acknowledges and values all gender identities.

- **Gender Identity**: One’s deeply felt internal experience of gender, which may or may not align with sex assigned at birth. It can reject fixed gender norms^2^.

- **Gender Equity**: Fairness and justice in distributing benefits and responsibilities among all genders. It is the means to achieve gender equality^3^.

- **Gender Equality**: Equal rights, responsibilities, and opportunities for all genders. It recognizes diversity and aims for inclusivity, not uniformity^2^. It’s a societal goal, not just a women’s issue.

- **Gender Gap**: Disparities between genders in areas like participation, rights, pay, and access^3^. While often measured between women and men, it applies to all gender identities.

- **Intersectionality**: A framework to understand how gender intersects with other identity factors (race, class, religion, etc.) and how these intersections create unique forms of discrimination^3^.

- **JEDI Principles**: Justice, Equity, Diversity, and Inclusion. Justice is seeking fairness in an unfair world. Equity provides equitable opportunities to everyone. Diversity is representation of different genders, races, ethnicities, sexual orientations, socioeconomic statuses, and religions. Inclusion actively promotes participation of all people. These guide efforts to address systemic inequities, promote fair access, and foster inclusive, respectful environments^4^.

- **Patriarchal Violence**: Gender-based violence rooted in systems of male dominance. It includes physical, sexual, psychological, or economic harm^3^, traditionally against women but also affecting other genders.

- **Violet Spot**: A Spanish initiative to combat gender-based violence by promoting awareness and providing resources^5^. Initially focused on women, it can be expanded to support all genders.

^1^ https://www.coe.int/en/web/gender-matters/glossary

^2^ https://www.unwomen.org/sites/default/files/2022-02/Handbook-on-gender-mainstreaming-for-gender-equality-results-en.pdf

^3^ https://eige.europa.eu/publications-resources/thesaurus

^4^ https://freshwater-science.org/justice-equity-diversity-inclusion-jedi-task-force

^5^ https://violenciagenero.igualdad.gob.es/informacion-3/puntovioleta/

## Characterizing the problem: gender bias in freshwater scientific societies

A close examination of freshwater scientific societies revealed that gender disparities persist within executive boards and that the presence of gender committees are far from common practice. We identified a total of 34 limnological societies across 32 different countries worldwide. First, we searched for information on the composition of each society’s executive board and the existence of a gender committee by reviewing the content available on their official websites. We included societies regardless of the language in which their websites or other public materials were available. When information was not available in English/Spanish/Portuguese, we translated the relevant content to ensure a consistent and inclusive assessment across all identified societies. Information about board members was publicly available for only 12 (35%) of the societies, and none of the websites provided information on the existence of a gender committee except for the AIL. To supplement these data, we sent a request for information to the societies’ institutional email address. If no response was received, we reached out to society members for whom we had contact details to request assistance. Through this process, we obtained information from 26 societies (76% of those identified) on the gender of the president, 24 societies (71%) on the composition of the board, and 12 societies (35%) on the existence of gender committees, respectively. We acknowledge that, in some cases, the recorded gender of the presidents and board members -based on names and pictures- may not reflect their self-identified or actual gender identity. However, due to the lack of more detailed information, this is the only data we had access to. While we recognize its limitations, it is the best available source for gender-related insights in this context. Data was collected between January and August 2024.

Most of the freshwater scientific societies we identified were found in Europe (45%), followed by Asia (18%) and South America (18%). The relatively low number of societies in Oceania and North America is likely due to the fact that, traditionally, European countries tend to have their own national societies, whereas in Oceania and North America larger, regional societies are common (e.g. Association for the Sciences of Limnology and Oceanography, ASLO; International Society of Limnology, SIL). In Africa, we identified only one freshwater scientific society, the Southern African Society of Aquatic Scientists, despite the continent comprising 54 countries and hosting some of the world’s most valuable freshwater ecosystems^16^. This limited presence might be related to lower levels of research funding, with much of the research conducted by foreign institutions that often perpetuate scientific colonialism^17^. In that context, establishing more scientific societies within Africa along with dedicated gender committees, even if initially composed by a few members^18^, can boost scientific collaboration among African researchers, and enhance the internationalization and recognition of their research.

**Fig. 1:**
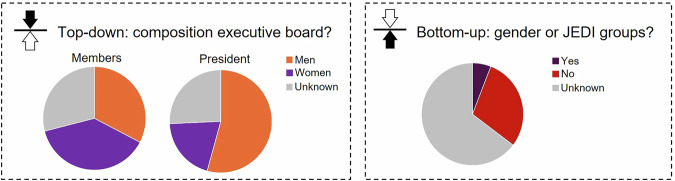
Percentage of men and women representation across president and board members, and presence of gender groups in freshwater scientific societies (n = 34). The percentage of societies with no available information is shown in grey.

Gender balance on the boards of freshwater scientific societies has been achieved in many of the cases for which we could gather information. In 46% of the societies consulted, women accounted for more than half of the board members; however, the presidency remained male-dominated (73% of the cases) (Fig. 1). Only the AIL and the Colombian Society (“Red Colombiana de Limnología” in Spanish) had a constituted gender committee, although four societies showed interest in its creation after consultation. Nonetheless, the North American Society of Freshwater Sciences (SFS) and the Society of Canadian Aquatic Sciences (SCAS) had a JEDI group, aimed at breaking down barriers for minoritised groups. Overall, out of the 34 limnological societies identified, we obtained information about gender committees for 12. Of these 12, only four had an active group working on inclusion and gender issues. This proportion of committees dropped from 33% to 12% if assuming that societies that did not respond to our request likely do not have gender or JEDI committees in place.

Scientific societies are typically smaller than universities and research centers, allowing the creation of a more friendly environment through regular conferences and direct interaction with board members. This less hierarchical structure often allows scientific societies more freedom to be inclusive compared to other academic or professional institutions (e.g. private companies). However, gender bias remains prevalent within these societies. Our findings suggest that, unfortunately, gender equality is not a priority for freshwater scientific societies since this information is generally lacking from their websites, gender and JEDI committees are absent from their structure, and presidencies are mainly dominated by men. The underrepresentation of women in leadership positions is a complex issue that extends beyond the scope of this paper, but research shows that this bias persists across many disciplines^19^. For instance, a study focused on zoological sciences found that while women held more leadership roles in these societies than in academic institutions (i.e. universities, research institutes), their representation was still low (~30%)^20^. Given the obvious gender bias in scientific societies, the presence of women on executive boards is strongly advised to ensure the participation of women in both the decision-making and social aspects (from internal governance to interactions with external stakeholders and society at large). Additionally, the existence of gender or JEDI committees within the scientific society can increase the visibility of women and other minoritised groups, support their work, and encourage their active involvement.

## A theory of change: ten steps to guide scientific societies towards gender equality

Scientific societies reflect local, social, cultural and political contexts and so have the opportunity to implement practices and protocols that promote gender equity and JEDI principles (see definition in Box 1). If JEDI is not operationalized it could disproportionately disadvantage groups historically excluded from science, such as women, LGTBIQ+ people (acronym that stands for Lesbian, Gay, Bisexual, Transgender, Intersex, and Queer/Questioning. The “+” includes other sexual orientations and gender identities, beyond those explicitly listed), non-white people, and people with disabilities. Overall, our goal is to raise awareness of the critical importance of embedding gender equity and JEDI principles in scientific societies, with the ambition of scaling it to academia at large, and to provide a theory of change to achieve this goal. We believe that fostering an inclusive environment built on mutual respect is vital for cultivating a diverse scientific community^21^. Beyond professional expertise, scientific societies can provide their members with access to supportive networks where empathy and emotional safety are prioritized. In scientific environments that can often feel hostile and overly competitive, a culture of inclusion helps individuals, especially those from marginalised groups, to contribute confidently, reach their full potential, and help create a better society and more meaningful careers^21^.

We envision a “Theory of Change to promote gender equality in scientific societies” that aims for an ideal, albeit utopian, scientific society that truly embodies and advances gender equality. This vision goes beyond supporting inclusivity: it calls for scientific societies to lead, advocate for, and drive meaningful change within their disciplines, by upholding JEDI principles in science. To generate real change, scientific societies must also advocate for public policies that support equity and inclusivity in research funding, hiring practices, and education. Although bottom-up efforts (such as volunteer groups) are often the starting point, lasting progress requires the engagement of executive boards and institutional leadership. Key actions, such as policy reforms and allocating financial resources to support equity, must ultimately come from top down. By influencing these broader structures, societies can help reshape academia into a more accessible and equitable science for all.

Our vision of an inclusive scientific society is captured in a ten steps guide designed to help societies develop gender-sensitive policies within their fields. These steps are inspired by the G&S-AIL group’s ten year experience (section “A decade of action: the gender group of the Iberian Society of Limnology”). The aim of this paper is to turn reflection into action. While our efforts have primarily focused on increasing the visibility of women in freshwater sciences and advocating for their rights, the experience gained and our *modus operandi* can be applied to any other minoritised group.

**Fig. 2:**
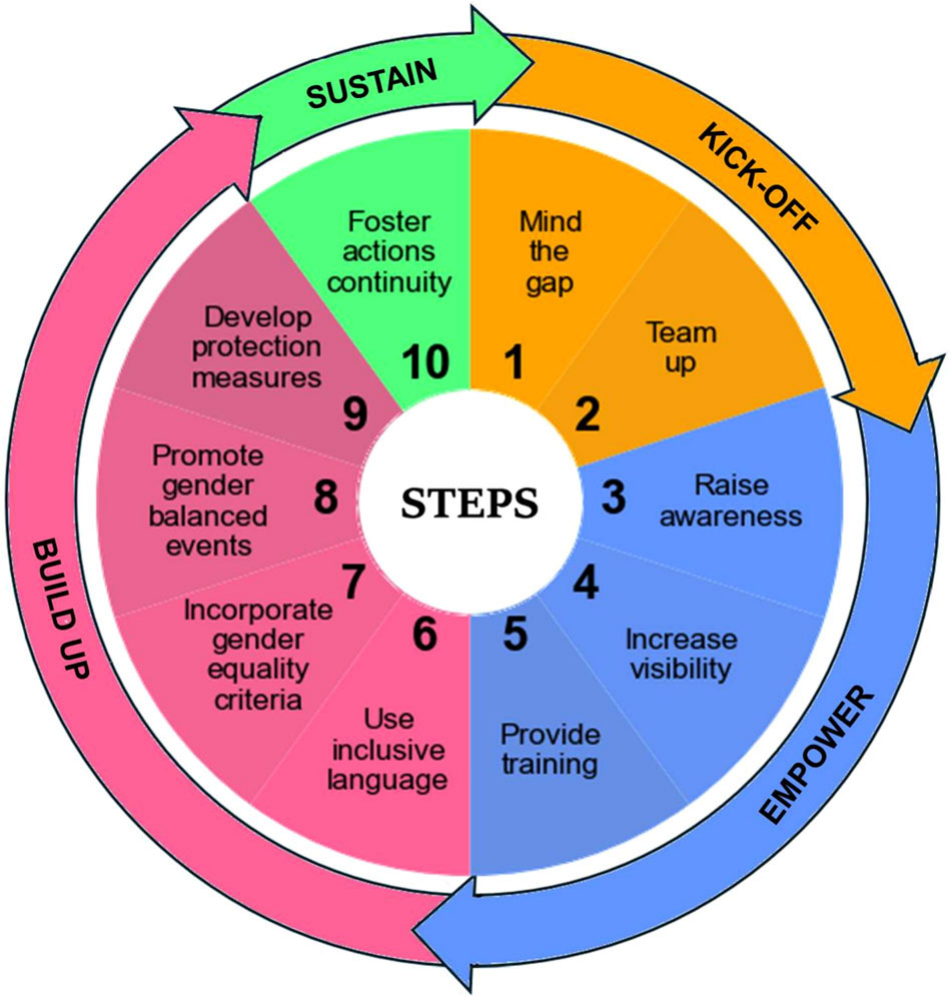
Diagram of the ten steps of the “Theory of Change to promote gender equality in scientific societies”coloured by the four defined phases.

The ten steps of this “Theory of Change to promote gender equality in scientific societies” are organised into four phases: *kick off* → *empower* → *build up* → *sustain* (Fig. 2). *Kick off* lays the groundwork by raising awareness; *Empower* spreads knowledge and tools; *Build up* focuses on putting this knowledge into practice; and *Sustain* ensures that efforts lead to lasting, structural change. The development of these phases is circular and iterative, as are the needs for change. For each step, we provide specific actions, with examples and resources to guide implementation. Actions are proposed to be mainly implemented by the society board, its gender or JEDI committees, or individual members. By adopting them, scientific societies can make meaningful progress toward a more inclusive, supportive community for people of all genders.

### KICK OFF phase

This phase builds the foundations for sustainable change by addressing gender gaps within the scientific society. It involves collecting and analyzing data on gender representation, identifying imbalances, and encouraging collaboration to promote equity. Early involvement of the executive board is key for effective and lasting impact. This phase is composed of two steps.

#### Step 1. Mind the gap

Identify and analyze gender gaps related to JEDI principles within the scientific society.

The first crucial step is to assess the current gender balance within the scientific society and recognize existing biases. This initial assessment provides a snapshot of a specific scientific discipline’s situation and a baseline to track changes over time. Actions include collecting demographic data, analyzing gender representation in leadership roles (such as authorship of conference abstracts and plenary speakers), and reviewing past conferences. Equally important is to engage members and the executive board through focus groups, surveys, and interviews to understand perceptions of gender bias and the implementation of JEDI principles. These analyses reveal disparities and member experiences, forming the foundation for addressing challenges faced by minoritised groups and guiding effective equity and inclusion strategies.

For instance, the G&S-AIL group assessed the status of women in freshwater sciences in the Iberian Peninsula by using the membership database of the AIL to analyze gender biases across different academic stages, reviewing plenary speaker data from past AIL conferences, and conducting a survey to capture members' perceptions on gender issues^22^. The idea of identifying gender gaps in a scientific society is closely linked to studies that highlight a gender productivity gap, which is primarily driven by a larger scientific output attributed to men^23^, pointing out that women are disadvantaged by citation metrics and are less likely to be authors of review papers which tend to receive more citations than original papers.

#### Step 2. Team up

Establish a dedicated committee within the scientific society that effectively advocates for gender equity and inclusivity.

After identifying gender biases, forming a dedicated gender committee within the scientific society is crucial to promote JEDI principles. This group provides a platform to address gender issues, amplify underrepresented voices, and foster an inclusive culture. Research highlights the value of workgroups and mentoring networking (early–career researchers mentored by experienced researchers) to build allyship and provide emotional support for women^24^. By uniting diverse perspectives, the committee can lead initiatives that embed gender diversity in the society, enhancing collaboration and driving more innovative research.

To successfully establish a gender (or JEDI) committee, it is essential to engage members and raise awareness on gender issues. The committee should be diverse, representing various genders, ethnicities, career-levels and social backgrounds, to enrich perspectives. Moreover, gender equity is a collective responsibility, not just for minority groups. Effective communication within the society and with the board is essential. The committee should create channels to keep everyone informed and appoint a representative to attend board meetings. Additionally, having a dedicated section on the society’s website can help share initiatives and reach a wider audience. For instance, the G&S-AIL group, a grass-root initiative of the AIL society members, initially analysed gender biases within their scientific society (Step 1^22^) and established itself as a committee within the society (Step 2), both steps naturally evolving in parallel. Over time, the group created a mailing list and a dedicated section on the AIL’s website (https://www.limnetica.com/en/genderscience), to share its history, objectives, projects, and activities. This platform also provides information on how to connect via email, mailing lists, subscriptions, and social media. Later, the group secured funding for an independent website (https://www.genderlimno.org) which now provides resources, project details, the group’s CV, a blog, and links to other initiatives aligned with the G&S-AIL group’s aims.

### EMPOWER phase

The goal of this phase is to create a knowledgeable and conscious community within the society that values and actively promotes gender equality. The *empower phase* aims to provide scientific societies and their members with both the skillset and knowledge to achieve this goal. The empower phase has three steps, which are steps three to five in the overall process.

#### Step 3. Raise awareness

Increase awareness among society members about gender biases, and garner the commitment of the scientific community and beyond.

This step focuses on recognizing and actively addressing gender biases by engaging the community. Specific actions include hosting seminars and sessions on gender bias in science, sharing data and reports that reveal existing disparities, and implementing mentorship programs and networking opportunities to foster inclusivity. One example is the British Ecological Society Mentoring Scheme which connects early-career ecologists, particularly those from minoritised groups, with senior mentors for guidance and inspiration^25^. Annual or biannual society meetings are ideal platforms for these initiatives, as they reach wide audiences. Integrating multidisciplinary approaches, such as combining STEM with art or social sciences, can enhance impact and accessibility. For example, the “Augmented ecofeminisms: climate, water and woman” exhibition, by the G&S-AIL group (section “A decade of action: the gender group of the Iberian Society of Limnology”), combines augmented reality with science and art to reflect how climate change affects freshwater ecosystems and women (https://www.genderlimno.org/muac.html).

#### Step 4. Increase visibility

Make women and other minoritised groups more visible within scientific societies and celebrate pioneering figures across different disciplines.

Visibility initiatives help address gender disparities by promoting recognition, inclusion in leadership roles, and the creation of role models for future generations^26,27^. Key actions include: (*i*) promoting achievements via society's newsletters, websites, conferences, and social media (e.g., STEM Women Congress, https://www.stemwomen.com, WISEcology conference https://www.wisecology.net/); (*ii*) creating directories of experts to showcase women scientists and support their inclusion in panels, media, and committees (e.g., 500 Women Scientist initiative https://500womenscientists.org/who-we-are); (*iii*) showcasing historically overlooked contributions from women and minoritised groups through exhibitions and curated lists (e.g., Women in Limnology and Key Figures in Limnology^28^ from G&S-AIL group); (*iv*) establishing awards named after influential women and scientists from other minoritised groups to address biases in academic recognition^29^; (*v*) importantly, visibility must go beyond symbolic gestures. Tokenistic inclusion can hinder real progress, so efforts must be grounded in a genuine commitment to equity and meaningful participation at all levels of the scientific community.

#### Step 5. Provide training

Creating educational resources and training programs on gender bias awareness helps bridge the gap between knowledge and practice, driving real change within the scientific community.

The specific actions include developing educational materials and/or platforms (such as articles, videos, and interactive tools) designed to address gender bias in scientific settings. Ensuring that these resources are easy to access encourages sustained engagement. For instance, the G&S-AIL group developed a higher education teaching package to increase the representation of women role models in geosciences lessons, alongside a self-evaluation survey on gender biases while teaching (https://www.genderlimno.org/limnoedu.html). Similarly, the “No more Matildas” initiative sheds light on historically overlooked female scientists and provides educational content to inspire future generations (see “Discover the findings of our Matildas”, https://www.nomorematildas.com). Likewise, the 11F International Day of Women and Girls in Science platform also provides materials to promote gender equity in STEM education (in Spanish: https://11defebrero.org/materiales-11f/). Other key actions include inviting experts in gender studies to lead training sessions addressed to society members to deepen understanding of implicit biases and its impact. Additionally, organizing workshops focusing on key areas such as biases in peer review, leadership, and career advancement, enables members to co-create practical solutions tailored to their society’s needs.

### BUILD UP phase

The *Build up* phase implements practical measures and actions that promote gender equity within scientific societies. This phase comprises four essential steps:

#### Step 6. Embrace inclusive language

Promote inclusive language within the society’s communications, publications, and events.

To promote diversity and avoid gender stereotypes, all society’s communications should use inclusive language that respects individuals' gender, race, ethnicity, sexual orientation, disability, and other characteristics. The key action is to create clear, society-specific language guidelines tailored to the scientific field and the linguistic diversity within the society. Existing inclusive language resources can serve as a starting point^30^, but customized guidelines help standardize terminology. Once established, a review process should be implemented for all communications, including publications, websites, newsletters, social media, and event materials. Notable examples include the American Psychological Association’s English-language guide^31^ and gender-inclusive guides published by the Spanish Women’s Institute^32^.

#### Step 7. Incorporate gender equity criteria for resources allocation

To reduce gender bias, scientific societies should integrate gender equity criteria into their grants, awards, scholarships, and publications. This includes establishing clear evaluation criteria to ensure a minimum 40% success rate for women and individuals of other gender identities (e.g., non-binary), and a maximum of 60% for men. Evaluation panels should reflect these equity goals. Proactive measures are encouraged, such as supporting applicants from historically excluded groups and funding initiatives that address structural barriers like subsidizing caregiving during conferences. Helpful resources include the United Nations guide for inclusive job postings^33^. Increasingly, institutions like the University of Michigan’s ADVANCE Program (https://advance.umich.edu/) and the Royal Society of Chemistry (https://www.rsc.org/) are adopting JEDI principles in resource allocation.

#### Step 8. Promote gender-balanced events

To advance gender equity in scientific conferences and other society events, it is crucial to prioritize inclusivity at all levels of event organization^14,34,35^. Specific actions include ensuring diverse representation among invited and plenary speakers, as well as striving for balanced representation within scientific committees. Academic conferences are crucial events for researchers’ networking and visibility, and diversity among plenary speakers can expand the range of role models for young researchers that will shape the future of the societies. Hosting plenary talks and special sessions on gender issues and JEDI principles can help raise awareness and foster discussions about equity in the scientific community. For example, the 2nd Iberian Society of Ecology (SIBECOL) Meeting (2022) featured a plenary talk addressing gender equality in academia, along with a special session dedicated to diversity and inclusion in Ecology. Both events were strategically scheduled on the first day of the conference to foster awareness and encourage meaningful discussions throughout the subsequent days of the meeting. Additionally, equitable management of speaking opportunities by chairs, along with the facilitation of work-life balance by offering family-friendly scheduling or caregiving support, can help remove potential barriers to women’s participation. Beyond women, accessibility must be prioritized by designing both physical and virtual event spaces to accommodate diverse needs. Finally, implementing and enforcing a clear code of conduct for events, requiring participants to acknowledge and adhere to it during registration, is absolutely essential to create a safe and welcoming environment for all attendees^36^. An example of a conference code of conduct can be found in (https://www.sibecol.org/mm/file/Code_of_Conduct.pdf)^37^.

#### Step 9. Develop protection measures

Establishing secure channels for reporting misconduct and clear action protocols is essential to ensure a safe and inclusive environment. These channels should enable both society members and non-members participating in society events to report instances of harassment, discrimination, or other inappropriate behaviour confidentially and without fear of retaliation. Violet spots (in Spanish, “Punto Violeta”) – designed contact points for reporting misconduct during scientific events (Box 1) – are an excellent example of such measures^38^. Furthermore, the scientific society should publicly reject hate speech and pseudoscientific discourse while actively supporting historically excluded groups, such as women and other gender identities. Clear action protocols should outline how reports will be handled, ensuring incidents are investigated, addressed, and resolved promptly, transparently, and fairly.

### SUSTAIN phase

This phase aims to ensure the long-term sustainability of gender equity efforts by embedding them into the scientific society’s structure and practices. Achieving lasting impact requires institutional support, ongoing planning, and regular progress evaluations to maintain gender inclusivity as a continuous priority. This phase consists of a single key step:

#### Step 10. Foster actions’ continuity

Ensuring the continuity of these steps is vital to achieving lasting gender balance in the scientific community supported by consistent funding, staffing, and regular content reviews. This helps institutionalize JEDI principles, provides visible role models for future generations, and ensures adaptability through ongoing evaluation of previous steps. To support the implementation and evaluation of these steps, it is important to regularly update them based on feedback and societal changes, formalize them into publicly available policies on the society’s website and social media, and build alliances with other scientific societies. Additionally, with the consolidation of a gender or JEDI committee, appointing a dedicated professional to manage the growing administrative workload is recommended. Several indicators can be used to evaluate progress in addressing gender bias: *i*) *gender representation and retention*: track the percentage of women and minoritised groups, as well as their retention rates within the society over time; *ii) advancement*: monitor the career progression of these groups, including promotions and tenure rate; *iii) funding distribution*: assess the allocation of research grants and funding awarded to women and underrepresented researchers compared to men; *iv) publication metrics:* examine authorship patterns in research publications to ensure equitable representation across genders in scientific outputs; *v) survey feedback*: conduct regular surveys to gather insights on the effectiveness of the implemented steps and encourage self-reflection; *vi) incident assessment*: compare the frequency and nature of reported incidents before and after implementing the previous steps to evaluate their effectiveness; *vii) gender-based participation rates:* monitor the gender distribution in various aspects of scientific conferences, including presentations, plenary sessions, and the scientific committee. Tracking these indicators over time helps identify trends and patterns, informing strategies to promote gender equity in academic events.

## A decade of action: the gender group of the Iberian Society of Limnology

**Fig. 3:**
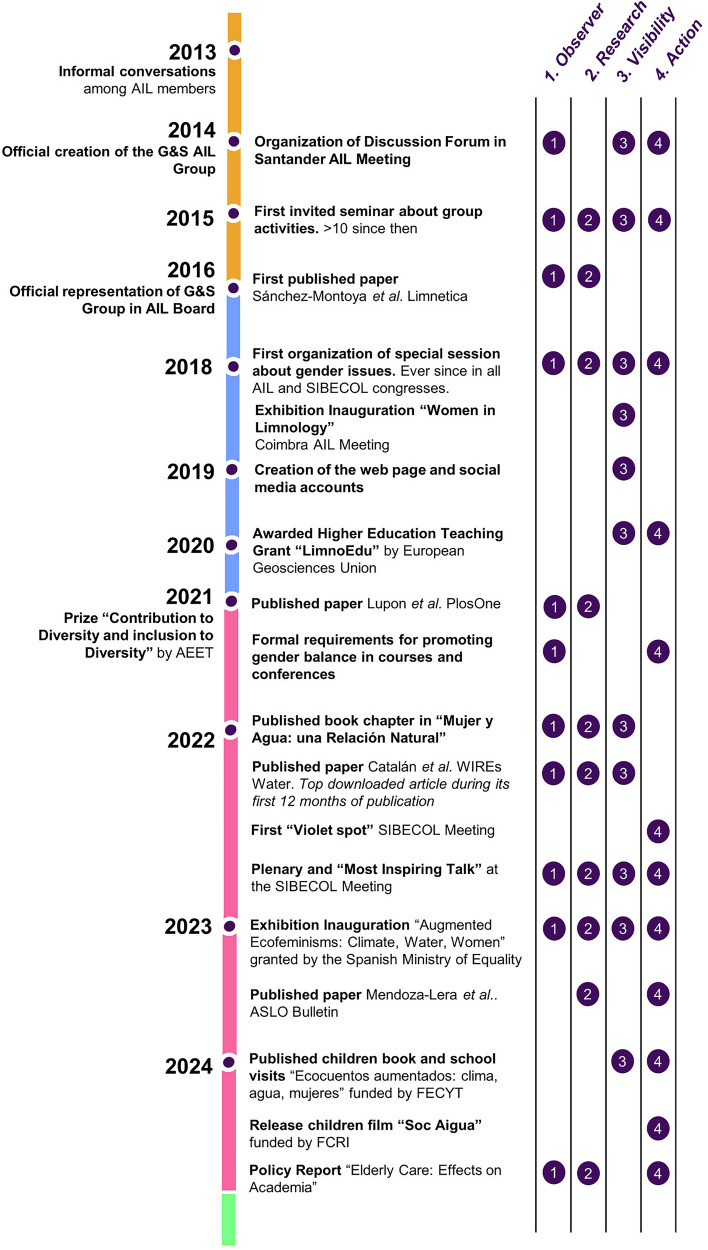
Timeline since the creation of the G&S-AIL group in 2014, highlighting the most relevant actions and the gender group objectives they addressed. The gradation of colour in the timeline illustrates the development of the different phases: *kick-off* (yellow) that corresponds to the first steps of establishing a gender group, *empower* and *build up* (in blue and pink, respectively) when the group and their activities are consolidated, expanded, and acknowledged by others, and *sustain* (green) that ensures that group efforts percolate through the community and last over time. See more details in section “Theory of change: 10 steps to guide scientific societies towards gender equality”.

The G&S-AIL group (https://www.genderlimno.org/) was created in 2014 within the Iberian Association of Limnology (AIL), and has had a representative within the AIL executive board since 2016. Importantly, we have received full support from the AIL board since the beginning, which has been crucial for developing our initiatives. At present, the group comprises more than 30 researchers from universities and research institutes mainly across the Iberian Peninsula. All members have diverse backgrounds in limnology but also terrestrial ecology and hydrology. We work voluntarily and collaboratively on projects and activities that aim to advance towards gender equality in academia, with a focus on freshwater sciences. The group has four main objectives: (1) to serve as an external *observer* of gender bias in our field, (2) to *research* gender issues, (3) to enhance the *visibility* of women in research within the scientific community and beyond, and (4) to promote *actions* to advance gender equity within AIL and academia (Fig. 3). Noteworthy, these objectives have evolved in parallel over time rather than sequentially (Fig. 3). While we acknowledge that gender bias affects people of all gender identities, our work has primarily focused on challenges faced by women researchers, which was the group’s original motivation. As the group has consolidated, we have broadened our perspective by exploring links between freshwater sciences and society, and to collaborate with other volunteer groups such as the Diversity & Inclusivity group of the Iberian Society of Ecology (SIBECOL) and the Diversity Committee of the Spanish Association of Terrestrial Ecology (AEET).

**Fig. 4:**
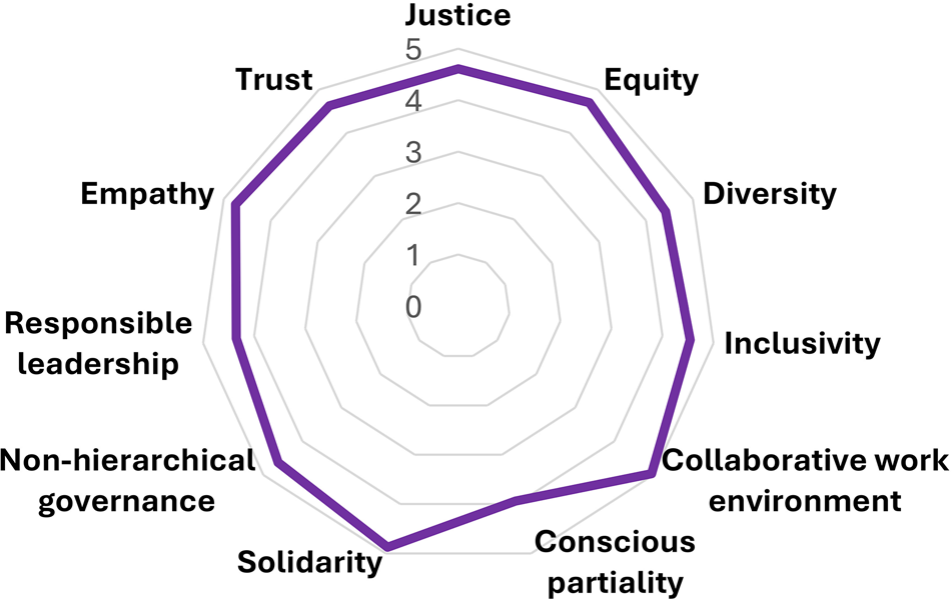
Spider plot synthesizing the answers to the question “Based on your experience, how much do you agree that the G&S-AIL group operates according to each of the following ethical principles?” included in the survey “Ethical Principles of the G&S-AIL group”. The values correspond to categories: 5 = Strongly agree; 4 = Agree; 3 = Neither agree nor disagree; 2 = Disagree; 1 = Strongly disagree: Line represents the mean values of answer received (*n* = 27). Definitions of the ethical principles can be found in Supplementary Information 1.

Most of our actions address the aforementioned objectives and align with the phases outlined in our ten steps guide. For example, our role as *observers* (first objective and present in most of our actions) was pivotal to our first internal discussion forum on gender biases within the scientific society at the 2014 AIL’s conference, which led to the creation of the gender committee (Fig. 3). Nearly half of our actions involve conducting research related to gender biases in freshwater sciences (objective 2), including publishing papers on barriers faced by women in their scientific careers and proposing measures to promote gender equity^22,28^. Actions aiming to increase the visibility of women in our discipline (objective 3) include the exhibition launched in 2018 ‘Women in Limnology’ (https://www.genderlimno.org/women-in-limnology.html), featuring panels and videos that highlight pioneering female freshwater scientists’ contributions. This exhibition is available for institutions to host. Finally, most of our *actions* have contributed to improve gender equity (objective 4) beyond academia. In 2024, we published a children’s book (https://www.genderlimno.org/aumenta.html) that illustrates freshwater environmental issues from a gender perspective, along with school visits to present it. The book also inspired a children’s film (https://www.rosercusso.com/soc-aigua). A key benefit derived from our work is the improved gender balance of invited talks during AIL conferences (women delivered 57% of plenary talks in 2020, up from 0% in 2006), and also the implementation of specific criteria to foster gender equity in AIL’s travel grants and scholarships (https://www.limnetica.com/en/projects-young-ail). However, challenges remain, such as ensuring gender balance in round tables and scientific courses organized by institutions outside AIL. Rather than take for granted the *modus operandi* and moral of our gender committee, we conducted an internal self-evaluation of our alignment with ethical principles (JEDI principles, collaborative work environment, conscious partiality, solidarity, non-hierarchical governance, responsible leadership, and empathy and trust). Between November and December 2024, we surveyed current and past volunteer members of the G&S-AIL group (see questions and definitions in SI1). The questionnaire assessed members’ perceptions of privileges, marginalization, and/or oppression within the group^39^, the member’s perception of collaborative moral practices, and the committee's commitment to the main JEDI principles, using a five-point Likert scale ranging from “strongly disagree” to “strongly agree”. This questionnaire and all the analyses therein were approved by the Ethical Research Committee of the Universidad Complutense de Madrid (CE_20240912_SOC_11). We encourage other gender and JEDI committees to carry out similar evaluations to reflect on and improve their internal practices. We sent the questionnaire to 61 researchers who have formed part of, or closely collaborated with the group, from which 27 responded. Most respondents were women (82%), mainly based in or from Spain (78%). On average, respondents had been involved in the gender committee for five years, ranging from one to 11 years. Most of the respondents (81%) currently participate in the group. Ages ranged from 31 to 67 years (median = 40), and 18% identified as LGTBIQ+. Most respondents (82%) work in academia, with over half on non-permanent contracts (59%). We acknowledge that the cultural and gendered contexts of our members significantly shape the perspective of the present manuscript. Moreover, this analysis highlights the need to engage more members of AIL, especially males, people from other minority groups, and early-career researchers to bring fresh perspectives and drive progress. All JEDI principles obtained average scores of at least 4 out of 5 (Fig. 4), suggesting that ethical principles are well-established within the committee. The highest scores were for “collaborative work environment” (4.9), which promotes kinder and more flourishing environments than competitiveness^40^, followed by “solidarity” (4.8), “empathy” (4.8), and “equity” (4.7). “Responsible leadership” scored slightly lower (4.4), likely reflecting our shared leadership approach. “Conscious partiality”, or the capacity to be completely unbiased or objective, scored the lowest (4.0), likely because members actively reflect on gender issues and acknowledge subjectivity. We plan to repeat this survey every five years to monitor trends and maintain a healthy, inclusive environment. Despite all our achievements, we acknowledge that there is still much work to be done.

## Conclusions and looking forward

Gender bias remains a significant issue in academia, and scientific societies can help close this gap by promoting equity and fostering inclusive, respectful environments. Our goal was to inspire scientific societies to pursue gender equity and better representation of minoritised groups, providing effective tools through the creation of internal gender or JEDI committees. For that, we showed in this manuscript our “Theory of Change to promote gender equality in scientific society” organised in ten steps to achieve our goal. Even in scientific societies, meant to uphold freedom and democracy, gender bias persists, and women rarely hold top leadership roles. While executive boards in freshwater scientific societies show some gender balance, fewer than one third have women presidents. Most strikingly, only four out of 34 societies have established gender or JEDI committees. This underscores the urgent need to constitute and support volunteer groups that champion JEDI values and raise awareness within the scientific societies. We believe that an utopian inclusive and gender-balanced academia is possible, and that this change can be catalyzed through gender and JEDI committees in scientific societies, which can be a great bottom-up strategy to foster these transformative changes. The abolitionist Mariame Kaba states that “hope is a discipline”. We must persevere, treating utopias as futures we must build and protect. This means persistence, self-evaluation, and a shared vision among scientific societies to exchange strategies and support. In a time when rights are being rolled back^41^ and free thought is under threat, inclusive academic communities can help defend those fundamental rights and amplify the voices of silenced minorities within and beyond academia.

## Supplementary information

Supplementary Information

## Data Availability

All data generated or analysed during this study are included in this published article [and its Supplementary Information files].

## Declaration of ai and ai-assisted technologies in the writing process

During the preparation of this work the authors used ChatGPT in order to improve readability and language. After using ChatGPT, the authors reviewed and edited the content as needed and take full responsibility for the content of the publication.

## References

[1] Iaria, A., Schwarz, C. & Waldinger, F. Gender gaps in academia: global evidence over the twentieth century. *SSRN* 4150221, 10.2139/ssrn.4150221 (2024).

[2] European Commission: Directorate-General for Research and Innovation. In *She figures 2021 – Gender in research and innovation – Statistics and indicators*, Publications Office, https://data.europa.eu/doi/10.2777/06090 (2021).

[3] Liu, S.-N. C., Brown, S. E. V. & Sabat, I. E. Patching the “leaky pipeline”: interventions for women of color faculty in STEM academia. *Arch. Sci. Psychol.* **7**, 32–39 (2019).

[4] Berhe, A. A. et al. Scientists from historically excluded groups face a hostile obstacle course. *Nat. Geosci.* **15**, 2–4 (2021).

[5] Kozolowsi, D. et al. Intersectional inequalities in science. *PNAS* **119**, e2113067119 (2022). 34983876 10.1073/pnas.2113067119 PMC8764684

[6] Grogan, K. How the entire scientific community can confront gender bias in the workplace. *Nat. Ecol. Evol.* **3**, 3–6 (2019). 30478306 10.1038/s41559-018-0747-4

[7] Dimitry, S. & Murphy, A. Beyond #MeToo: the gender equity iceberg, https://www.academia.edu/35921937/Beyond_MeToo_The_Gender_Equity_Iceberg (2017).

[8] Greenaway, F. *Science international: a history of the international council of scientific unions* (Cambridge University Press, 1996).

[9] Abernethy, E. F. et al. Diverse, equitable, and inclusive scientific societies: progress and opportunities in the Society for Freshwater Science. *Freshw. Sci.* **39**, 363–376 (2020).

[10] Carroll, M. A., Boynes, S., Jerome-Majewska, L. A. & Topp, K. S. The imperative for scientific societies to change the face of academia: Recommendations for immediate action. *Anat. Rec.* **305**, 1019–1031 (2022). 10.1002/ar.24735 34418322

[11] Primus, C. et al. Scientific societies fostering inclusivity in the life sciences through engagement of undergraduate scientists. *Front. Educ.* **7**, 757816 (2022).

[12] Shiffman, D. S. et al. What can professional scientific societies do to improve diversity, equity, and inclusion: A case study of the American Elasmobranch Society. *Front. Educ.* **7**, 842618 (2022).

[13] Spirito, F. et al. Gender stereotypes in ecological research themes: an analysis of the last 20 years of the Argentinian ecology conferences. *Austral Ecol.* **49**, e13301 (2024).

[14] Lupon, A. et al. Towards women-inclusive ecology: Representation, behavior, and perception of women at an international conference. *PLoS One* **16**, e0260163 (2021). 34890389 10.1371/journal.pone.0260163 PMC8664204

[15] Segarra, V. A. et al. Scientific societies fostering inclusive scientific environments through travel awards: current practices and recommendations. *CBE Life Sci. Educ* **19**, es3 (2020). 32453676 10.1187/cbe.19-11-0262 PMC8697665

[16] Shumway, C. A. Forgotten waters: freshwater and marine ecosystems in Africa. In *Strategies for biodiversity conservation and sustainable development* (Boston University, 1999).

[17] Seth, S. Putting knowledge in its place: science, colonialism, and the postcolonial. *Postcolonial. Stud.* **12**, 373–388 (2009).

[18] Gaëta, B. A. et al. Ten simple rules for forming a scientific professional society. *PLoS. Comput. Biol.* **13**, e1005226 (2017). 28333920 10.1371/journal.pcbi.1005226 PMC5363797

[19] Howe-Walsh, L. & Turnbull, S. Barriers to women leaders in academia: tales from science and technology. *Stud. High. Educ.* **41**, 415–428 (2016).

[20] Potvin, D. A., Burdfield-Steel, E., Potvin, J. M. & Heap, S. M. Diversity begets diversity: a global perspective on gender equality in scientific society leadership. *PLoS One* **13**, e0197280 (2018). 29847591 10.1371/journal.pone.0197280 PMC5976142

[21] Tsui, A. S. From traditional research to responsible research: the necessity of scientific freedom and scientific responsibility for better societies. *Annu. Rev. Organ. Psychol. Organ. Behav.* **9**, 1–32 (2022).

[22] Sánchez-Montoya, M. M. et al. Women in limnology in the Iberian Peninsula: biases, barriers and recommendations. *Limnetica* **35**, 61–72 (2016).

[23] Astegiano, J., Sebastián-González, E. & Castanho, C. D. T. Unravelling the gender productivity gap in science: a meta-analytical review. *R. Soc. Open Sci.* **6**, 181566 (2019). 31312468 10.1098/rsos.181566 PMC6599789

[24] Grunwald, S. & Daroub, S. A 360° perspective of women in soil science focused on the U.S. *Front. Soil Sci.* **3**, 1072758 (2023).

[25] Young, D. M., Rudman, L. A., Buettner, H. M. & McLean, M. C. The influence of female role models on women’s implicit science cognitions. *Psychol. Women Q.* **37**, 283–292 (2013).

[26] Chikere, C. B. et al. Research collaborations for enhanced performance and visibility of women scientists. In *Science by women: stories from careers in STEM* (eds Karr, R. P., Hultgren, C. L. & Welch, K. M. A.) 43–59 (Springer, 2022).

[27] Damschen, E. I. et al. Visibility matters: increasing knowledge of women's contributions to ecology. *Front. Ecol. Environ.* **3**, 212–219 (2005).

[28] Catalán, N. et al. Women in limnology: from a historical perspective to a present-day evaluation. *Wiley Interdiscip. Rev. Water* **10**, e1616 (2023).

[29] Gehmlich, K. & Krause, S. How we name academic prizes matters. *Nat. Hum. Behav.* **8**, 190–193 (2024). 37989864 10.1038/s41562-023-01773-9

[30] Soon, Z. & Owens, M. Inclusive Language Guide–Communication in HealthCare, Science, Education and the Workplace. Anatomy and Physiology Review. British Columbia/Yukon Pressbooks, https://pressbooks.bccampus.ca/anatomyandphysiologyreview/ (2024).

[31] American Psychological Association. Inclusive language guide, 2nd ed. https://www.apa.org/about/apa/equity-diversity-inclusion/language-guidelines.pdf (2023).

[32] Instituto de las Mujeres. Guías para el uso no sexista del lenguaje. Instituto de la Mujer y para la igualdad de oportunidades (Spanish Government). https://www.inmujeres.gob.es/servRecursos/formacion/GuiasLengNoSexista/docs/Guiaslenguajenosexista_.pdf (2017).

[33] UN Women. Guidance on Creating Inclusive Vacancy Announcements: Good Practice Examples from the UN. The Office of the Focal Point for Women in the UN System at UN Women, https://www.unwomen.org/sites/default/files/Headquarters/Attachments/Sections/How%20We%20Work/Gender-parity/Gender-parity-Vacancy-announcements-good-practices-en.pdf (2022).

[34] Hall, S. M. et al. Ten simple rules for pushing boundaries of inclusion at academic events. *PLoS Comput. Biol.* **20**, e1011797 (2024). 38427633 10.1371/journal.pcbi.1011797 PMC10906823

[35] Débarre, F., Rode, N. O. & Ugelvig, L. V. Gender equity at scientific events. *Evol. Lett.* **2**, 148–158 (2018). 30283672 10.1002/evl3.49 PMC6121837

[36] Favaro, B. et al. Your science conference should have a code of conduct. *Front. Mar. Sci.* **22**, 10.3389/fmars.2016.00103 (2016).

[37] SIBECOL Code of conduct. https://www.sibecol.org/mm/file/Code_of_Conduct.pdf (2023).

[38] Blanco-Fuente, I., Blanco-García, M. E., Martín-Peláez, P., Peláez-Orero, S. & Romero-Bachiller, C. *Violet spots against sexual harassment in the University: an activist collective response from Spain*, 37. (EASST Review, 2018).

[39] SFS JEDI Task Forced. Reflecting on our privileged and marginalized identities in SFS. [Conference presentation]. SFS Annual Meeting, Virtual, https://freshwater-science.org/sites/default/files/medialib/sfs_2021_-_jedi_engagement_1-_05.25.2021_.pptx.pdf (2021).

[40] Schumann, F. et al. Beyond kindness: a proposal for the flourishing of science and scientists alike. *R. Soc. Open Sci.* **10**, 230728 (2023). 38026042 10.1098/rsos.230728 PMC10663797

[41] Leifer, A. M., Liu, A. J. & Nagel, S. R. US researchers must stand up to protect freedoms, not just funding. *Nature* **641**, 592–593 (2025). 40360893 10.1038/d41586-025-01466-5

