# Supplementary material for: Breaking barriers: ten essential steps to achieve gender equality in academia through scientific societies
Source: NPJ Biodivers. 2025 Oct 1;4:37. doi: 10.1038/s44185-025-00105-6 (PMC12488902; doi:10.1038/s44185-025-00105-6)
Supplement: Supplementary file 1 — Supplementary Information [file 44185_2025_105_MOESM1_ESM.docx]

**SUPPLEMENTARY INFORMATION 1**

**Questionnaire**

The questionnaire includes aspects about the ethical principles of the Gender & Science AIL (Iberian Association of Limnology) group (G&S-AIL group, <https://www.genderlimno.org/>; <https://www.limnetica.com/en/genderscience>), since its establishment in 2014. This questionnaire is an evaluation about the main ethical principle that guides this group. The target audience is researchers who have taken part in any period since 2014 being involved in any of the different activities developed by the G&S-AIL group (around 70 ecology and social researchers).

The questionnaire will take around 10 minutes to be completed.

**QUESTIONNAIRE “ETHICAL PRINCIPLES OF GENDER & SCIENCE AIL GROUP”**

**Window 1 – Informed consent**

You are invited to respond to this questionnaire about the ethical principles of the Gender & Science AIL (Iberian Association of Limnology) group (hereafter, G&S-AIL group) based on your experience collaborating in different activities (such as projects, publications and exhibitions). Principles included in the questionnaire are: justice, equity, diversity, inclusivity, conscious partiality, solidarity, non-hierarchical governance (horizontality rather than verticality), responsible leadership, empathy and trust. The questionnaire has been developed by the G&S-AIL group. If you have any questions about the questionnaire, please contact [genderscienceail@gmail.com](mailto:genderscienceail@gmail.com).

We would like to save your responses to include them in a research study about promoting actions towards gender equity through scientific societies. To do so, we will ask you some basic demographic questions. This questionnaire is anonymous, and your responses will be kept completely confidential. Non personally identifiable data will be collected and no one will be able to link your answers back to you. Participation in this research is completely voluntary. If you decide to participate, you may withdraw at any point during the study, for any reason, and without any prejudice. By clicking the "I consent, I wish to participate in the study" button below, you acknowledge that your participation in the study is voluntary, you are 18 years of age or older, and that you are aware that you may choose to terminate your participation in the study at any time and for any reason. If you prefer to not participate in the study, you can take the self-assessment questionnaire and your responses will not be saved in our server.

- I consent, I wish to participate in the study

- I do not wish to participate in the study but I want to take the assessment questionnaire

- I do not with to participate in the study neither to take the assessment questionnaire

**Window 2 - BLOCK 1: INFORMATION FOR ANALYSIS** - Part A

To start, we just need some information about yourself. Please, mark the appropriate
answers.

**Q01.** What´s your age?

- Drop down (*a drop-down menu is a closed question that presents a drop-down list of options*)

**Q02.** What is your gender?

- Female

- Male

- Different gender identity (please specify):

**Q03.** Which country is your nationality?

- Drop down

**Q04.** In which country are you currently living?

- Drop down

**Q05.** Do you belong to any underrepresented minority group? Select all that qualify.

- Race/ethnic minority

- LGBT (lesbian, gay, bisexual, transgender)

- People with disabilities

- Other (please specify):

- None

**Q06.** In which career stage are you?

- Undergraduate or Master Student

- PhD Candidate / Pre-doctoral Researcher

- Early Career Researcher / Post-doctoral Researcher

- Senior Professor/Researcher - Non-permanent position

- Senior Professor/Researcher - Permanent position

- Other (please specify):

**Window 3 - BLOCK 1: INFORMATION FOR ANALYSIS** - Part B

We need to know information about your experience with the G&S-AIL group. Please, mark the appropriate answers.

**Q07.** How many years did you participate in any activity of the G&S-AIL group?

- Drop down (less than 1 year; 1; 2; 3; 4; 5; 6; 7; 8; 9; 10; 11)

**Q08.** Do you currently participate in the G&S-AIL group?

- Yes

- No

**Window 4** - BLOCK 2. GENERAL INFORMATION ABOUT ETHICAL PRINCIPLES

**Q09.** Based on your experience in the G&S-AIL group, how much do you agree that the group operates according to each of the following ethical principles? 5-level Likert scale for each aspect:

- Strongly disagree

- Disagree

- Neither agree or disagree

- Agree

- Strongly agree

***Definitions**

**1. JEDI principles (justice, equity, diversity, and inclusion)**.

*Justice:* is defined as dismantling barriers to resources and opportunities in society so that all individuals and communities can have the same experience.

*Equity:* recognizes that advantages and barriers exist and is defined as allocating resources to ensure that everyone has access to the same opportunities.

*Diversity:* is defined as all the differences between us based on which we experience advantages or encounter barriers to opportunities (recognizing differences).

*Inclusivity:* is defined as promoting and sustaining a sense of belonging; it values and practices respect for the talents, beliefs, backgrounds, and ways of living of its members.

**2. Collaborative work environment:** is defined as the promotion of teamwork and cooperation, in which participants can work together towards a common goal, rather than against each other. This can help reduce the pressure and stress associated with competition and foster a more supportive work environment.

**3. Conscious partiality:** is defined as the creation of a dialectical distance between the researcher and participant, and it enables the correction of distortions of perception on both sides and widens the consciousness of both, the researcher and the researched.

**4. Solidarity:** is defined as mutual support irrespective of status, gender or ethnicity.

**5.** **Non-hierarchical governance:** is defined as the fact of promoting horizontal rather than vertical governance structures, by having few centralized authorities with few management levels.

**6. Responsible leadership:** is a mindset characterized by a personal sense of normative, ethical, and moral obligations and leaders are willing to be held accountable for the consequences of their actions and behaviors.

**7. Empathy and trust:** are defined as the ability to understand, and are essential for creating a positive work culture where individuals feel valued and appreciated.

**Q10.** Which of the previously listed ethical principles is the most important for you?

- Opened response

**Q11.** Is there any other ethical principle not listed above that is important for you?

- Opened response
